# Case Report: autologous stem cell boost enables hematopoietic recovery after severe cytopenia induced by BCMA-targeted bispecific antibody therapy in multiple myeloma

**DOI:** 10.3389/fonc.2025.1705843

**Published:** 2026-01-05

**Authors:** Hiroaki Akiyama, Seiji Kakiuchi, Akimasa Sakamoto, Naru Tomigaki, Shutaro Fujioka, Isamu Harima, Ryotaro Niwa, Ikumi Takagi, Yoko Kozuki, Yoshiharu Miyata, Kyoko Yoshihara, Satoshi Yoshihara, Nobuko Iwata

**Affiliations:** 1 Department of Hematology, Yodogawa Christian Hospital, Osaka, Japan; 2 Department of Artificial Intelligence and Digital Health Science, Kobe University Graduate School of Medicine, Kobe, Japan; 3 Department of Hematology, Hyogo Medical University, Nishinomiya, Japan

**Keywords:** BCMA, bispecific antibody, cytopenia, elranatamab, hematopoietic recovery, multiple myeloma, stem cell boost

## Abstract

Elranatamab, a bispecific antibody targeting B-cell maturation antigen (BCMA) and CD3, has shown significant efficacy in relapsed or refractory multiple myeloma (RRMM). However, elranatamab therapy also carries a risk of hematologic toxicity. Stem cell boost (SCB), involving reinfusion of previously collected autologous CD34^+^ hematopoietic stem cells, has been explored as a salvage strategy after Chimeric Antigen Receptor T-cell (CAR T-cell) therapy-related cytopenias. To date, its role following bispecific antibody treatment remains unclear. We report the case of a 59-year-old man with RRMM who developed prolonged pancytopenia after elranatamab therapy, successfully rescued with autologous SCB. Neutrophil recovery occurred by day +10, transfusion independence by day +19, and hematologic recovery allowed elranatamab resumption without recurrence of severe cytopenia. This case demonstrates the potential utility of SCB for managing persistent cytopenias after T-cell–redirecting therapies and supports the importance of early stem cell collection.

## 1 Introduction

Relapsed or refractory multiple myeloma (RRMM) continues to represent a significant clinical challenge, particularly in patients who have previously been exposed to proteasome inhibitors, immunomodulatory agents, and anti-CD38 monoclonal antibodies. Bispecific antibodies targeting B-cell maturation antigen (BCMA) and CD3, including elranatamab, have demonstrated promising efficacy in heavily pretreated RRMM populations. The MagnetisMM-3 trial reported an overall response rate of 61%, with manageable toxicity profiles, although hematologic adverse events such as grade ≥3 neutropenia (49%) and thrombocytopenia (24%) were frequently observed (1). Prolonged cytopenias, particularly in patients with prior hematopoietic injuries, have emerged as a significant clinical concern (2, 3).

Despite the expanding role of BCMA-directed bispecific antibodies, optimal management strategies for treatment-emergent, persistent cytopenias remain undefined. Stem cell boost (SCB), involving reinfusion of autologous cryopreserved CD34^+^ hematopoietic stem cells, has been employed as a salvage strategy in the post-CAR T-cell setting. It is particularly indicated in cases of immune effector cell-associated hematotoxicity (ICAHT) (4–7, 10). Several retrospective studies have shown that SCB facilitates multilineage hematologic recovery within weeks to months, without apparent adverse effects on disease progression or survival outcomes (4, 6, 7, 10). Although SCB has been increasingly utilized as a rescue strategy for immune effector cell-associated hematotoxicity following CAR T-cell therapy, evidence supporting its role after BCMA–CD3 bispecific antibodies remains limited. In contrast to CAR T-cell therapy, where the relationship between inflammatory cytokine activation and marrow suppression is well described, the mechanisms underlying prolonged cytopenias after bispecific antibodies and their optimal management are still unclear.

To our knowledge, detailed reports describing successful hematopoietic recovery with SCB that allowed safe resumption of elranatamab have not been published. Therefore, this case extends the current literature by demonstrating that SCB may be a feasible salvage option for patients experiencing severe, persistent cytopenias induced by BCMA-directed bispecific antibodies.

Herein, we describe a patient with RRMM who developed severe, prolonged pancytopenia following elranatamab administration, successfully managed with autologous SCB. This case illustrates the potential utility of SCB in reversing bispecific antibody-associated cytopenias and supports its consideration as a therapeutic approach for selected patients receiving novel T-cell–engaging immunotherapies.

## 2 Case presentation

A 59-year-old man was diagnosed in 2016 with IgG λ-type multiple myeloma, classified as International Staging System stage III. He was considered eligible for high-dose chemotherapy with autologous stem cell transplantation (HDCT/ASCT) and received four cycles of bortezomib, cyclophosphamide, and dexamethasone, followed by four cycles of bortezomib and dexamethasone. He then underwent high-dose cyclophosphamide-primed stem cell mobilization and collection, yielding 7.7 × 10^6^ CD34^+^ cells/kg, which were cryopreserved in preservation bags at −80 °C.

During pre-transplant evaluation, a left renal mass was incidentally detected. He underwent left nephrectomy, and histopathological examination confirmed renal cell carcinoma. Because of subsequent solitary kidney status, HDCT/ASCT was not performed due to the potential nephrotoxicity of high-dose melphalan.

**Figure 1:**
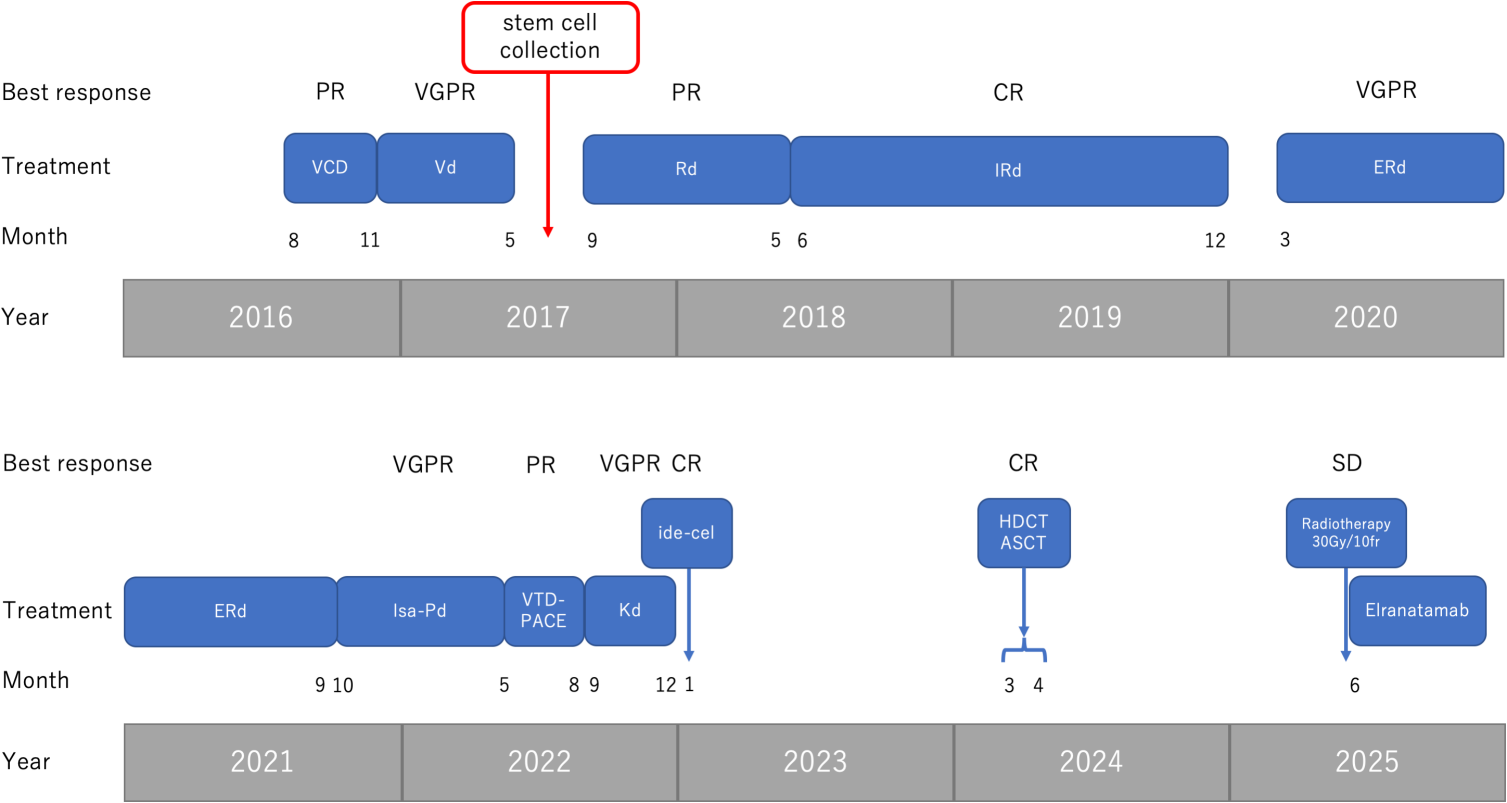
Overview of treatment history and clinical responses from 2016 to 2025. The figure summarizes all major therapeutic lines administered during the disease course, including VCD, Vd, Rd, IRd, ERd, Isa-Pd, VTD-PACE, Kd, ide-cel (BCMA-CAR T-cell), high-dose chemotherapy with autologous stem cell transplantation (HDCT/ASCT), radiotherapy, and elranatamab. For each regimen, the corresponding best clinical response (PR, VGPR, CR, SD) is shown along the timeline. Key clinical events—including stem cell collection, disease relapse, and initiation of elranatamab—are also indicated. Abbreviations: VCD, bortezomib-cyclophosphamide-dexamethasone; Vd, bortezomib-dexamethasone; Rd, lenalidomide-dexamethasone; IRd, ixazomib-lenalidomide-dexamethasone; ERd, elotuzumab-lenalidomide-dexamethasone; Isa-Pd, isatuximab-pomalidomide-dexamethasone; VTD-PACE, bortezomib-thalidomide-dexamethasone-cisplatin-doxorubicin-cyclophosphamide-etoposide; Kd, carfilzomib-dexamethasone; ide-cel, idecabtagene vicleucel; HDCT/ASCT, high-dose chemotherapy with autologous stem cell transplantation; PR, partial response; VGPR, very good partial response; CR, complete response; SD, stable disease.

Over the following years, he received multiple lines of therapy for RRMM, including proteasome inhibitors, immunomodulatory drugs, and anti-CD38 antibody-based regimens (**Figure 1**).

In January 2023, he received idecabtagene vicleucel (ide-cel) CAR T-cell therapy. He achieved disappearance of M-protein on immunofixation and minimal residual disease (MRD) negativity. In November 2023, M-protein reappeared, though 18F-fluorodeoxyglucose positron emission tomography (PET) revealed no disease sites. In December 2023, 11C-methionine PET demonstrated multiple focal uptakes in the spine, sternum, pelvis, femurs, and humeri, and persistent M-protein confirmed relapse.

Given relapse after CAR T-cell therapy and the absence of a prior autologous transplant, HDCT/ASCT was selected as the next therapeutic approach. As part of the pre-transplant evaluation, bone marrow cytogenetics (G-banding) demonstrated 46,XY,del (20)(q11.2q13.3) in 3/20 metaphases, with no morphologic features of myelodysplastic syndrome. Because nearly eight years had passed since the initial harvest, the patient underwent repeat mobilization with filgrastim 400 μg/m² daily for six days and plerixafor 0.24 mg/kg daily for two days, yielding 1.0 × 10^6^ CD34^+^ cells/kg. In March 2024, he underwent ASCT; conditioning consisted of high-dose melphalan with a 20% dose reduction for renal impairment (total 140 mg/m² administered over two days), followed by infusion of the newly collected 1.0 × 10^6^ CD34^+^ cells/kg. Neutrophil engraftment was achieved on day +17, but cytopenias persisted (grade 3 anemia and grade 4 thrombocytopenia).

By April 2025, he achieved transfusion independence, although grade 2 anemia and grade 3 thrombocytopenia remained. In June 2025, he developed lumbar pain, and CT revealed an L1 vertebral mass compressing the nerve root. Blood tests revealed detectable M-protein, and bone marrow examination showed 10% plasma cells, confirming relapse. The lumbar mass observed on CT was also considered a myeloma lesion. He received local radiotherapy (30 Gy in 10 fractions) and corticosteroids for symptom control, followed by the initiation of elranatamab.

However, after the first dose, he developed grade 3 anemia and grade 4 thrombocytopenia, leading to discontinuation of therapy. Bone marrow biopsy revealed marked hypocellularity without detectable myeloma cells, consistent with elranatamab-related toxicity.

Due to persistent cytopenias and the inability to resume elranatamab, autologous SCB was planned using stem cells collected in 2017, which at the time of collection contained 7.7 × 10^6^ CD34^+^ cells/kg. Prior to infusion, post-thaw enumeration confirmed a total of 4.6 × 10^6^ viable CD34^+^ cells/kg. In July 2025, this product was infused via peripheral venous access over approximately 20 minutes without additional conditioning chemotherapy. Premedication included acetaminophen and an antihistamine. Supportive care consisted of daily subcutaneous filgrastim at 300 μg/m² starting on day 0, antibacterial and antifungal prophylaxis, and transfusions according to institutional thresholds (red blood cell transfusion for hemoglobin <7 g/dL and platelet transfusion for platelet count <20 × 10^9^/L). Complete blood counts were monitored daily until hematologic recovery.

**Figure 2:**
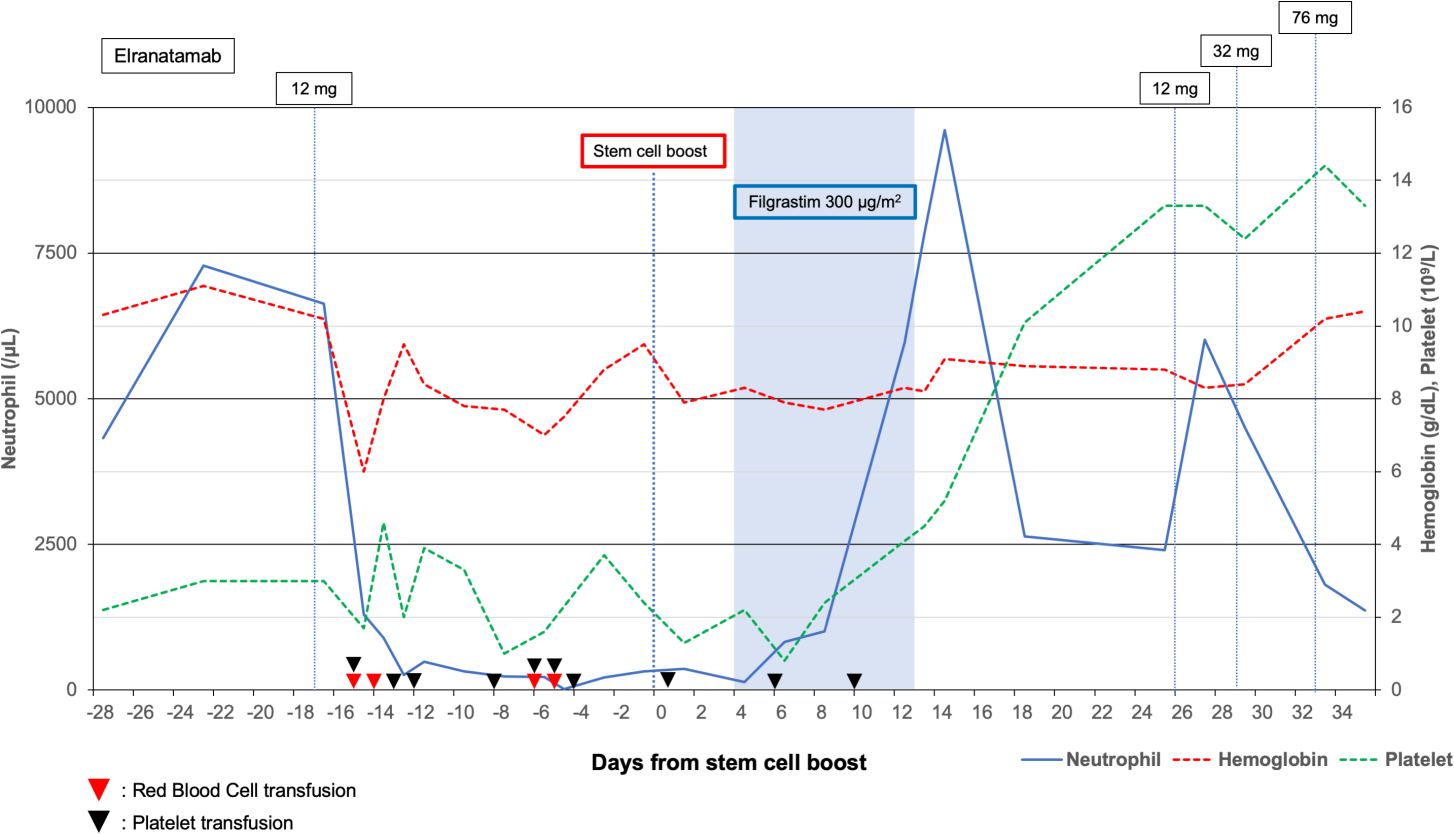
Treatment timeline around elranatamab administration and autologous stem cell boost (SCB). Annotations indicate filgrastim 300 μg/m² administration and elranatamab step-up dosing (12 mg, 12 mg, 32 mg, 76 mg). Symbols denote transfusions: red triangle, red blood cell transfusion; black triangle, platelet transfusion.

Neutrophil engraftment was achieved on day +10 (defined as the day of absolute neutrophil count ≥0.5 × 10^9^/L for three consecutive days), and platelet transfusion independence was reached by day +19. Hemoglobin and platelet counts gradually improved thereafter. Elranatamab was resumed only after the patient met predefined safety criteria, including an ANC ≥1.0 × 10^9^/L, platelet count ≥50 × 10^9^/L, hemoglobin ≥8 g/dL without transfusion, absence of any active infection, and improvement in marrow cellularity. A cautious step-up dosing regimen was reintroduced, and complete blood counts were monitored three times per week during the first three weeks of re-exposure. No recurrence of severe cytopenias was observed, allowing the continuation of therapy. Peripheral blood count trends following elranatamab administration and SCB are shown in **Figure 2**. After hematologic recovery and successful reinitiation of elranatamab, follow-up CT demonstrated stable disease in the L1 vertebral lesion. Despite the limited radiological response, serum M-protein became undetectable, and bone marrow evaluation confirmed the complete disappearance of clonal plasma cells.

## 3 Discussion

In this report, we describe a case of RRMM in which elranatamab was administered after multiple prior treatments, including various chemotherapy regimens, ASCT, and CAR T-cell therapy. Although current expert consensus generally recommends switching to non-BCMA-targeted agents after failure of a BCMA-directed therapy (11), in our patient, elranatamab was selected because of limited alternative options, the absence of available clinical trials, and the urgent need for disease control. This case suggests that sequential anti-BCMA therapy may still provide clinical benefit in carefully selected patients, although its efficacy is likely to be reduced and requires further investigation (12).

Elranatamab treatment was interrupted due to prolonged cytopenias; however, administration of an autologous SCB using previously collected stem cells resulted in hematologic recovery, enabling resumption and continuation of elranatamab therapy. Prolonged cytopenias following T-cell–redirecting therapies, including bispecific antibodies and CAR T-cell therapy, are well-recognized adverse events in RRMM. To our knowledge, this is the first reported case in which SCB successfully managed elranatamab-induced prolonged cytopenias, highlighting its potential role as a salvage strategy in similar clinical scenarios.

Prolonged cytopenias following CAR T-cell therapy, often referred to as ICAHT, have been reported in up to 20–40% of patients, with grade ≥3 cytopenias persisting beyond 60 days in a substantial proportion (3, 8). The pathophysiology is multifactorial, involving cumulative marrow injury from prior therapies, cytokine-driven suppression during immune activation, and disruption of the bone marrow microenvironment (13). Notably, bispecific antibodies such as elranatamab and teclistamab exhibit similar hematologic profiles, with grade ≥3 neutropenia in approximately 40–50% and thrombocytopenia in 30–40% of treated patients (1, 9). These effects are often amplified in heavily pretreated patients, especially those previously exposed to CAR T-cell therapy or autologous transplantation, as exemplified by our patient.

Management of prolonged cytopenias is critical, as they may limit the feasibility of ongoing treatment. Patients face heightened risks of severe infection and bleeding, prolonged hospitalization, and chronic transfusion dependence (14). More importantly, cytopenias frequently lead to treatment delays or discontinuation, which can compromise disease control and increase the risk of early relapse (15). In our patient, elranatamab showed an initial response but required immediate interruption after the first dose due to life-threatening cytopenia. Without an effective rescue strategy, such interruptions would lead to morbidity such as severe infections, bleeding, and hospitalizations, resulting in increased mortality. Autologous SCB offers an effective and practical salvage strategy in patients with prolonged cytopenias after bispecific antibody therapy. SCB involves reinfusion of previously collected CD34^+^ cells without conditioning chemotherapy, thereby restoring hematopoietic progenitors and facilitating marrow recovery. Retrospective studies in post-CAR T-cell populations demonstrate that 80–90% of patients achieve meaningful recovery of neutrophil and platelet counts within 2–4 weeks (4–7, 10). Our patient achieved neutrophil engraftment by day +10 and transfusion independence by day +19, consistent with prior reports—including those where SCB was performed after prolonged cytopenias following BCMA-directed CAR T-cell therapy.

Adequate preservation of stem cells is essential for performing an SCB; therefore, determining the optimal timing of stem cell collection is of critical importance. While an adequate number of CD34^+^ cells (7.7 × 10^6^/kg) was collected and cryopreserved in 2017, a subsequent attempt to collect stem cells at the time of ASCT in 2024—after prior CAR T-cell therapy and multiple lines of treatment—yielded only 1.0 × 10^6^/kg. This experience highlights that the mobilization potential of hematopoietic stem cells may decline significantly after extensive cytotoxic and cellular therapy, emphasizing the importance of early collection when feasible (16). In the evolving era where CAR T-cell and bispecific antibodies are increasingly applied earlier in the treatment course, the early collection and preservation of autologous stem cells provide a critical safety net for patients at risk of therapy-related marrow failure. This strategy may be particularly valuable in heavily pretreated patients or those with limited hematopoietic reserve.

Although the infused CD34^+^ cell dose (1.0 × 10^6^/kg) was below the generally recommended threshold for optimal engraftment (≥ 2.0 × 10^6^ CD34^+^/kg), previous reports have shown that engraftment can still be achieved with doses in this range, often with slower hematologic recovery and prolonged anemia or thrombocytopenia (17, 18). Therefore, the ASCT procedure performed in this case was considered reasonable given the limited stem cell yield after multiple prior intensive therapies, although cytopenias persisted for several months. Prior reports indicate that even after more than a decade of cryostorage, CD34^+^ cell counts are largely preserved and marrow engraftment is not adversely affected (19, 20). Given that the use of long-term cryopreserved stem cells is generally regarded as safe and effective, utilizing the product collected in 2017 may have been an appropriate option for ASCT. Consistent with these observations, in our case, the product cryopreserved in 2017 was thawed, and subsequent enumeration confirmed an adequate viable CD34^+^ cell dose for infusion. The stem cells had been cryopreserved for more than seven years yet retained sufficient viability to support hematopoietic recovery, underscoring the long-term utility of early stem cell collection and storage.

Looking ahead, further research is required to optimize the timing and patient selection for SCB. Prospective studies could clarify the minimal CD34^+^ cell dose required, and evaluate long-term outcomes, including immune reconstitution and infection risk.

Nevertheless, this report has several limitations. First, it describes a single patient, and therefore its findings cannot be generalized. Second, the prolonged cytopenias observed in our case were likely multifactorial, reflecting the cumulative impact of prior cytotoxic chemotherapy, CAR T-cell therapy, and a suboptimal stem cell dose at the time of salvage ASCT. The low CD34^+^ cell dose (1.0 × 10^6^/kg) is known to be associated with delayed multilineage recovery, and prior CAR T-cell therapy may have induced long-term alterations in the bone marrow microenvironment, including stromal dysfunction and inhibitory cytokine signaling. In addition, the presence of del(20q), although not accompanied by morphologic dysplasia, may indicate reduced hematopoietic reserve. Despite these contributing factors, the abrupt onset of grade 4 thrombocytopenia and marked marrow hypocellularity immediately after the first elranatamab dose strongly suggests that bispecific antibody-related toxicity was the dominant driver of marrow suppression, superimposed on an already compromised hematopoietic system.

Finally, the follow-up period after SCB is relatively short, and longer observation will be necessary to confirm the durability of hematopoietic recovery and ongoing disease control.

In conclusion, our case illustrates that autologous SCB represents a practical yet underutilized intervention for severe, prolonged cytopenias following BCMA–CD3 bispecific antibody therapy. SCB can reduce transfusion burden, allow therapy continuation, and ultimately contribute to improved outcomes in patients treated with advanced cellular immunotherapies for multiple myeloma.

## Data Availability

The original contributions presented in the study are included in the article/supplementary material. Further inquiries can be directed to the corresponding author.
